# Active constituent of *Polygala tenuifolia* attenuates cognitive deficits by rescuing hippocampal neurogenesis in APP/PS1 transgenic mice

**DOI:** 10.1186/s12906-021-03437-5

**Published:** 2021-10-25

**Authors:** Xiao-feng Wang, Hong-he Xiao, Yu-tong Wu, Liang Kong, Ji-cong Chen, Jing-xian Yang, Xiao-le Hu

**Affiliations:** 1 Center for Neuromedicine of Dalian Municipal Central Hospital, 42 Xuegong Street, Shahekou District, Dalian, Liaoning Province 116033 People’s Republic of China; 2 School of Pharmacy, Liaoning University of Traditional Chinese Medicine, 77 Life One Road, DD Port, Dalian, Liaoning Province 116600 People’s Republic of China

**Keywords:** Alzheimer disease, Neurogenesis, Neural stem cells, Polygala tenuifolia, 3,6′-disinapoyl sucrose

## Abstract

**Background:**

Alzheimer’s disease (AD) is the most common dementia worldwide, and there is still no satisfactory drug or therapeutic strategy. *Polygala tenuifolia* is a traditional Chinese medicine with multiple neuroprotective effects. In present study, we investigated the effects of three active constituents [3,6′-disinapoyl sucrose (DISS), onjisaponin B (OB) and tenuifolin (TEN)] of *Polygala tenuifolia* (PT) on the proliferation and differentiation of neural stem cells (NSCs) to identify the potential active constituent of PT promoting hippocampal neurogenesis.

**Methods:**

NSCs were isolated from hippocampi of newborn C57BL/6 mice, and transfected with mutant amyloid precursor protein (APP) gene to establish an AD cell model (APP-NSCs). 3-(4,5- Dimethylthiazol-2-yl)-2,5-diphenyltetrazolium bromide (MTT) and lactate dehydrogenase (LDH) assays were performed, and the proliferation and differentiation of NSCs were assessed by neurosphere formation assay, 5-bromo-2′-deoxyuridine (BrdU) incorporation assay and immunofluorescence (IF) staining analysis. APP/PS1 transgenic mice were administrated with the potential active constituent DISS for 4 weeks. Morris water maze (MWM), Nissl staining assay and IF staining assays were carried out to evaluate the cognitive function, neural damages and hippocampal neurogenesis, respectively.

**Results:**

DISS exerted the optimal ability to strengthen APP-NSCs proliferation and neuronal differentiation, followed by OB and TEN. Furthermore, DISS treatment for 4 weeks strikingly rescued the cognitive deficits, neuronal injures, and neurogenesis disorder in adult APP/PS1 transgenic mice.

**Conclusions:**

Our findings demonstrated that DISS is the constituent of PT that triggers the most potent increase of hippocampal neurogenesis in our mouse model of AD.

**Supplementary Information:**

The online version contains supplementary material available at 10.1186/s12906-021-03437-5.

## Background

Alzheimer’s disease (AD) is a neurodegenerative disorder associated with extensive symptoms, including progressively declines in language, cognitive and orientation functions, which result in dysfunction in personality and behavior [1]. Currently, AD is insulting more than 50 million individuals worldwide. Moreover, along with the aggravated aging of world’s population, the risk of developing AD increases, which will lead to a tremendous socio-economic challenge. Unfortunately, no drugs or agents are available to treat or to prevent the disease right now [2]. AD is characterized by neuropathological hallmarks, including neurofibrillary tangles (NFTs), senile plaques (SPs) formed by amyloid beta (Aβ) accumulation, chronic neuroinflammation, synapse loss and neuronal death [1, 3, 4]. In addition, dysfunction of hippocampal neurogenesis has been identified in both AD patients and AD rodent models [5–7].

Adult hippocampal neurogenesis is the process that continually generates new neurons from neural stem cells (NSCs) in the hippocampus, and plays is a vital role in hippocampal plasticity and cognitive function [8]. It has been well demonstrated that adult hippocampal neurogenesis persists throughout the lifetime in mammals and human beings [9]. However, this process decreases with aging and neurodegenerative diseases including AD. New born neurons are still detectable in AD patients, the amount is severely lower than that in age-matched healthy controls [10–12]. A growing body of literature indicates that deficits of hippocampal neurogenesis are already observed at a pre-symptomatic stage of AD, which further aggravate the cognitive disorder of this disease [5–7]. Thus, extensive efforts are underway to exploit feasible approach to stimulate endogenous neurogenesis in the hippocampus of AD brains. Fortunately, compounds such as metformin and β-asarone which can modulate stages involving proliferation, migration, and differentiation of NSCs development would promote hippocampal neurogenesis to ameliorate cognitive function in adult rodents [13, 14].

*Polygala Tenuifolia* (PT), also known as Yuan Zhi in Chinese, is a famous traditional Chinese medicine which has been widely applied to treat central nervous system diseases including dementia and neurasthenia [15]. Numerous pharmacological studies have demonstrated that PT possessed multiple neuroprotective effects associated with AD, such as anti-apoptosis [16], anti-neuroinflammation [17], enhancing neurotrophy [18] and anti-neurotoxicity induced by β-amyloid (Aβ) [19]. Moreover, it has been reported that PT extract promoted NSCs proliferation in vitro and enlarged the amount of NSCs in hippocampal CA1 regions in adult rats [20]. Phytochemical studies have revealed that there are more than 100 compounds including saponins, xanthones, and oligosaccharide esters in PT [21]. Onjisaponin B (OB), 3,6′-disinapoyl sucrose (DISS) and Tenuifolin (TEN) are the major active constitutes of PT. Previous studies have revealed that OB could mitigate the cognitive impairments by elimination of neuroinflammation, oxidative stress and Aβ pathology in AD animals [22, 23]. DISS could protect SH-SY5Y cells from apoptosis induced by glutamate [24, 25]. TEN is a metabolite of OB [26], and it has been demonstrated that oral administration of TEN markedly improved the cognitive function of AD animals [27, 28]. However, the potential active constituent of PT to promote the proliferation of NSCs and whether it could promote hippocampal neurogenesis in APP/PS1 mice are still relatively unexplored.

In present study, using the NSCs overexpressed amyloid precursor protein (APP-NSCs) as the AD cell model [29, 30], we sought to investigate the potential beneficial effects of the three active constituents of PT mentioned above on the survival, proliferation and differentiation of APP-NSCs in vitro, and further evaluate the effects of the potential active constituent DISS on the hippocampal neurogenesis in adult APP/PS1 transgenic mice.

## Materials and methods

### Chemicals and reagents

**Fig. 1:**
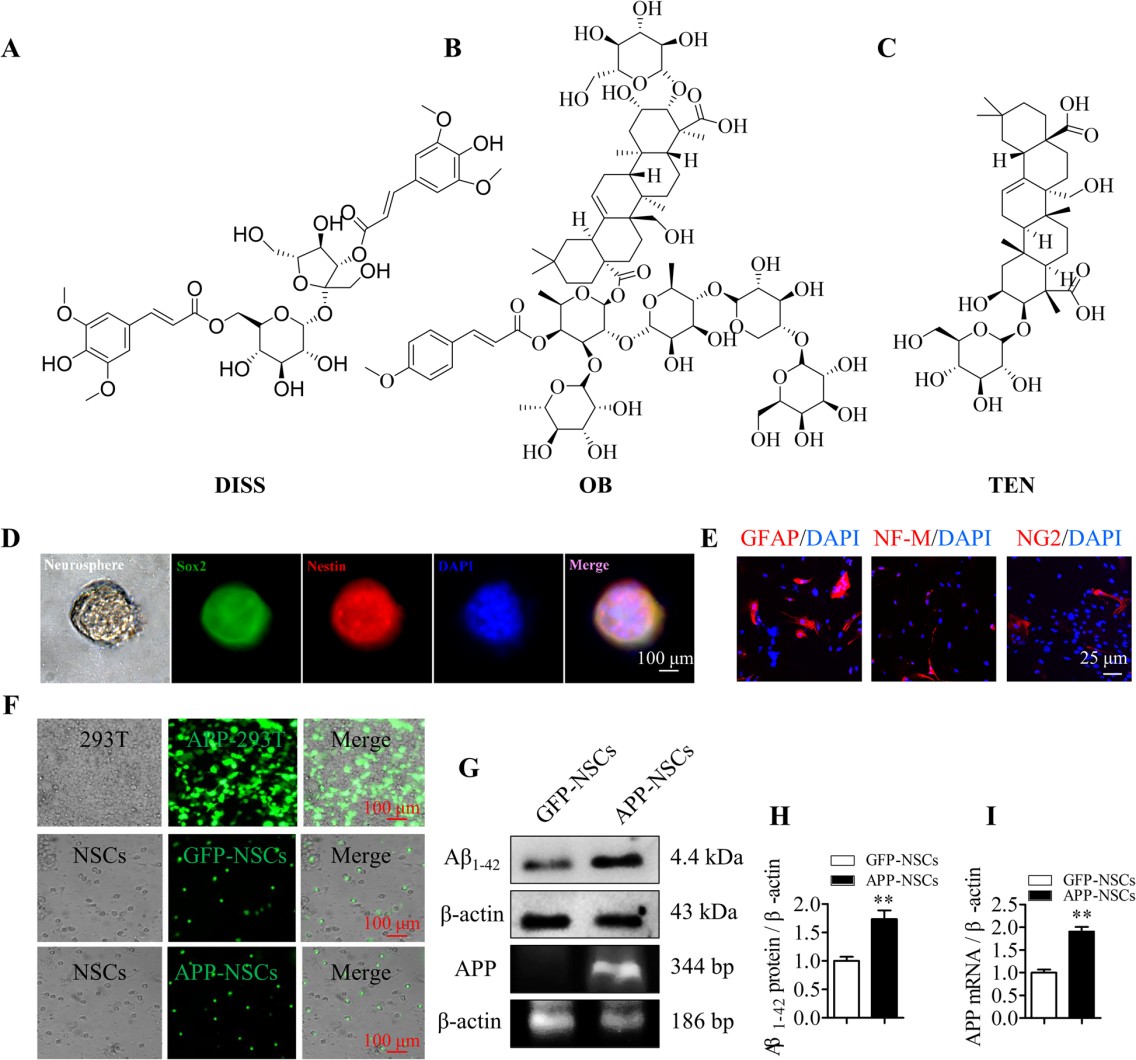
Chemical constructions of the three compounds, NSCs culture and AD cell model establishment. Chemical constructions of the three compounds. (A) 3,6′-disinapoyl sucrose (DISS); (B) onjisaponin B (OB); (C) tenuifolin (TEN). Hippocampal cells were isolated from newborn C57BL/6 mice; and cultured in proliferation medium for 7 days to obtain neurospheres; and then dissociated the neurospheres into single cells cultured in differentiation medium for 7 days to test the multi-directional differentiation ability. To establish AD cell model; the neurospheres were transfected with APP gene; and RT-PCR and Western blot analysis were used to determine the transfection. (D) The NSCs were double positive for Sox-2 (green) and Nestin (red); Scale bar = 100 μm; (E) The neurons; astrocytes and oligodendrocytes were marketed by NF-M; GFAP and NG2; respectively. Scale bar = 25 μm. (F) The 293 T cells and NSCs transfected with APP-GFP and GFP plasmids expressed green fluorescent protein. Scale bar = 100 μm; (G) The Aβ protein and mRNA levels in GFP-NSCs and APP-NSCs were measured by RT-PCR and Western blot assay; respectively; (H-I) Semi-quantification of Aβ protein (H) and mRNA (I) as in G. **P < 0.01 vs. GFP-NSCs group. Data are represented as mean ± sem. Data were analyzed by t-test

The 3,6′-disinapoyl sucrose (DISS, Lot: JOT10848, purity> 98%), Onjisaponin B (OB, Lot: JOT10575, purity> 98%) and Tenuifolin (TEN, Lot: JOT10162, purity> 98%) were purchased from the Chengdu Pufei De Biotech Co., Ltd. (Chengdu, China). The 5-bromo-2′-deoxyuridine (BrdU, Lot: MB3126–2) was purchased from Dalian Meilun Biotechnology Co., Ltd. (Dalian, China). Dulbecco’s modified Eagle’s medium (DMEM)/F12 medium, B27 supplement (Lot: 17054–044), non-essential amino acid (NEAA, Lot: 11140050), and GlutaMAX supplement (Lot: 35050061) were obtained from Gibco. Fetal bovine serum (FBS) was obtained from Invitrogen (Carlsbad, CA, USA). Basic fibroblast growth factor (bFGF, Lot: 100-18C) and epidermal growth factor (EGF, Lot: 315–09) were purchased from PeproTech company (Suzhou, China). The lactate dehydrogenase (LDH) kit was purchased from Nanjing Jiancheng Biotechnology, Co. (Nanjing, China). The primary antibodies including Rabbit anti Nestin (Lot: bs-20607R), Rabbit anti glial fibrillary acidic protein (GFAP, Lot: bs-0199R), Rabbit anti intermediate neurofilament (NF-M, Lot: bs-0710R), and Mouse anti BrdU (Lot: bs-0917R) were purchased from Beijing Bioss Biotechnology Co., Ltd. (Beijing, China). Mouse anti sex-determining region Y-box 2 (Sox-2, Lot: GT1876), Rabbit anti neural/glial antigen-2 (NG-2, Lot: PA5–27452), and Mouse anti neuron-specific nuclear protein (NeuN, Lot: MA5–33103) were purchased from Invitrogen (Carlsbad, CA, USA). The secondary antibodies labeled Cy3 or fluorescein isothiocyanate (FITC) were purchased from Jackson ImmunoResearch Inc. (West Grove, PA, USA). The chemical constructions of the three compounds were as shown in Fig. 1 A-C.

### Animals

APP/PS1 mice were purchased from the Model Animal Resource Information Platform (Nanjing, China). The littermate wild-type (WT) male C57BL/6 mice were used as normal control. Mice were aged about 9-month-old at the time of experiments (*n* = 10 in each group). All the mice were acclimated on a condition with access to mouse chow and water for 7 days before experiments. The temperature was 23 ± 2 °C, the humidity was 65 ± 5%, and the illumination was 12 h light/12 h dark cycle. New-born C57BL/6 mice were used for isolation and culture of NSCs in vitro. All experiments were approved by the Animal Core and Welfare Committee of Liaoning University of Traditional Chinese Medicine (use license number: SYXK (Liao) 2019–0004). All animal experiments complied with the ARRIVE guidelines and were carried out in accordance with Guide for the Care and Use of Laboratory Animals published by the US National Institutes of Health (NIH Publication No. 85–23, revised 1996).

### NSCs culture and AD cell model establishment

The NSCs culture and APP genes transduction were carried out as described in our previous studies [30, 31]. Briefly, the hippocampal cells isolated from new-born C57BL/6 mice were cultured in proliferation medium for 7–10 days. The proliferation medium consists of DMEM/F 12 medium supplemented with 1% non-essential amino acid (NEAA), 2% B 27, 1% GlutaMAX, 20 ng/ml EGF, 20 ng/ml bFGF and 0.5% penicillin/streptomycin. To identify the cultured NSCs, the neurospheres were underwent immunofluorescence staining (IF) against Nestin and Sox-2, respectively. To assess the multipotential differentiation, the cultured NSC suspensions were cultured in differentiation medium for 7 days, followed by IF staining against GFAP, NF-M and NG2, respectively. The differentiation medium consists of DMEM/F12 medium supplemented with 1% NEAA, 2% B27, 1% GlutaMAX, 1% fetal bovine serum (FBS) and 0.5% penicillin/streptomycin.

The green fluorescent protein (GFP) and APP-GFP plasmids were obtained from Professor Ya-Ping Yan in Shaanxi Medical University. To achieve AD cell model, NSCs were transfected with APP-GFP lentiviral vector and GFP lentiviral vector, respectively, as described in our previous studies [30]. Three days later, the transfection efficiency was evaluated using by RT-PCR and Western blot analysis.

### Cell viability

The cell viability was detected using by MTT assay. Briefly, single NSC suspension was seeded into 96-well plates at a density of approximately 1 × 10^6^ cells/ml, cultured in proliferation medium for 24 h. Then, cells were exposed to DISS (20, 40 and 60 μM), OB (2.5, 5 and 10 μM) and TEN (20, 40 and 60 μM), respectively, for another 24 h. After that, 10 μL MTT (5 mg/mL) solution was added into each well, and incubated for 4 h in the CO_2_ incubator. Replace the culture medium with 100 μL dimethyl sulfoxide to dissolve the formazan. The absorbance was measured at 532 nm using a microplate reader, and the cell viability of control group was normalized as 100%.

### LDH assay

Single NSC suspension was seeded into 96-well plates at a density of approximately 1 × 10^6^ cells/ml, cultured in proliferation medium for 24 h. Then, cells were exposed to DISS (20, 40 and 60 μM), OB (2.5, 5 and 10 μM) and TEN (20, 40 and 60 μM), respectively, for another 24 h. After that, the culture medium was harvested for the detection of LDH using a LDH kit according to the manufacturer’s instruction.

### Neurosphere culture

As described previously [32], the GFP-NSCs and APP-NSCs suspensions were seeded into 24-well plates at a density approximately of 1 × 10^5^ cells/ml, and cultured in proliferation medium in the presence or absence of DISS (60 μM), OB (10 μM) and TEN (60 μM) for 7 days, respectively. The diameters of resulting neurospheres in different groups were measured using Image J software.

### BrdU labeling

The GFP-NSCs and APP-NSCs suspensions were seeded into a 96-well plate coated with poly-D-lysine at a density approximately of 1 × 10^6^ cells/ml, cultured in proliferation medium in the presence or absence of DISS (60 μM), OB (10 μM) and TEN (60 μM) for 24 h, respectively. Cells were incubated within 20 μM of BrdU for 12 h to label proliferating cells, followed by IF analysis [32].

### NSCs migration assay

Neurospheres about 200 μm in diameter were plated into 24-well plates coated with poly-D-lysine, and cultured in differentiation medium. The neurospheres were exposed to 60 μM of DISS, 10 μM of OB and 60 μM of TEN, respectively. After 3 days’ exposure, pictures of the neurospheres were captured under an inverted microscope. Image-J software was applied to measure the migration distance of neural cells from the edge of the neurospheres [33].

### NSCs differentiation assay

The GFP-NSCs and APP-NSCs suspension were plated into 96-well plates in proliferation medium overnight. Replaced the proliferation medium with the differentiation medium supplemented with or without DISS (60 μM), OB (10 μM) and TEN (60 μM), cultured in the incubator for 7 days, followed by IF analysis against GFAP (glial fibrillary acidic protein, astrocyte marker), NF-M (intermediate neurofilament, neuron marker), and NG2 (neural/glial antigen 2, oligodendrocyte marker) to investigate the effects of the three compounds on the differentiation of NSCs.

### IF staining assay for culture cells

IF staining analysis was performed as described previously [14]. In short, cells were fixed with 4% paraformaldehyde (PFA) at 4 °C for 20 min, permeabilized with 0.5% Triton X-100 at room temperature (RT) for 20 min, blocked with 5% bovine serum albumin (BSA) for at RT for 60 min, incubated with primary antibodies against GFAP (1:150), NF-M (1:200), NG2 (1:200) and BrdU (1:150) at 4 °C overnight, respectively. Cells were rinsed with PBS for 3 times, incubated with Cy3 or FITC-conjugated secondary antibodies at RT for 60 min, counterstained with DAPI for 10–15 min to label the nuclei.

As to double-staining against Sox-2 and Nestin, cells were incubated within primary antibody mixture composed by mouse anti-Sox-2 (1:150) and rabbit anti-Nestin (1:150) for 18–24 h, rinsed with PBS for 3 times, followed by incubation with secondary antibody mixture composed by Cy3-conjugated goat anti-rabbit and FITC-conjugated donkey anti-mouse secondary antibodies at RT for 60 min.

As to BrdU staining, before blocking with 5% BSA, cells were exposed to 2 M of HCl at 37 °C for 30–40 min, rinsed with borate buffer (pH = 8.5) for 3 times to neutralize the HCl. The images were captured under random visual fields using an inverted fluorescence microscope (Nikon Eclipse E 800, Tokyo, Japan).

### Animals and drug administration

DISS was prepared with 0.3% sodium carboxymethyl cellulose (CMC-Na) into solutions with concentrations of 0.5, 1 and 2 mg/mL. Male APP/PS1 mice aged 9-month-old were randomly divided into the following 4 groups (*n* = 10 per group): model control group (APP/PS1), DISS-Low group (5 mg/kg), DISS-Middle group (10 mg/kg) and DISS-High group (20 mg/kg). The dose of DISS was determined according to the preliminary screen and previous study [34]. Another 10 littermates of wild-type male C57BL/6 mice were taken as normal control (WT). Mice in DISS treatment groups were orally administrated with different doses of DISS mentioned above once a day for 28 days. Mice in APP/PS1 group and WT group were orally given 0.3% CMC-Na as vehicle.

### Morris water maze (MWM) test

The MWM experiments were performed as described previously [35, 36]. In short, a circular tank filled with 40 cm depth of water (21 ± 1 °C) containing non-toxic white paint was used to perform the MWM experiments. In the training trials, individual mouse received two trainings per day for 5 consecutive days, and the escape latency was measured to evaluate the spatial acquisition capacity. After the last training trial, the probe test was performed. The mouse swam in the tank without escape platform for 120 s, the platform crossing numbers and the time spent in the target quadrant were recorded to evaluate the memory ability.

### Tissue preparation

After the MWM test, mice were sacrificed, perfused with cold saline to rinse the blood, followed by 4% PFA to fix the tissues. The brain tissues were dehydrated within 30% sucrose at 4 °C for 18–24 h, embedded within optimal cutting temperature embedding medium, coronally sectioned into 10 μm sections, stored at − 80 °C until usage [37]. The hippocampal areas were checked in the Nissl staining assay and IF staining assays.

### Nissl staining assay

Nissl staining assay was carried out to investigate the morphological impairments of neurons in the hippocampal regions according to the specification. In short, brain sections were fixed with 4% PFA at 4 °C for 20 min, washed with cold PBS for 3 times, stained with Nissl staining solution (C0117; Beyotime Biotechnology) for 5 min, rinsed with 95% ethanol for 5 min, transparentized within dimethylbenzene, sealed with neutral gum. Microscopic images were captured using the Nikon Eclipse E 800 microscope (Nikon, Tokyo, Japan). Image J software was applied to count the number of Nissl bodies.

### IF staining assay for brain tissues

Brain sections were fixed with cold PFA for 30–40 min, permeabilizated with 1% Triton X-100 at RT for 30 min, followed by blocking with 5% BSA at RT for 60 min. Sections were subsequently incubated with primary antibodies including mouse anti-Sox-2 (1:150), rabbit anti-Nestin (1:200), and mouse anti-NeuN (1:200), respectively, at 4 °C for 18 h. Then, sections were rinsed with PBS for 3 times, incubated with Cy3 or FITC-conjugated secondary antibodies at RT for 1.5 h, incubated with DAPI for 10 min to counterstain nuclei. The entire hippocampal regions including CA1, CA3 and dentate gyri (DG) regions were scanned.

### Statistical analysis

Data were presented as the mean ± sem. One-way ANOVA with Tukey post tests was applied to evaluate differences among multiple groups. Unpaired Student’s *t* test was applied to examine differences between two groups. Repeated-measures two-way ANOVA was used in the escape latency data in the MWM test. *P*-value less than 0.05 (*P <* 0.05) was considered statistically significant [38].

## Results

### NSCs culture and AD cell model establishment

After being cultured in proliferation medium for 7–10 days, the neural cells isolated from hippocampi of newborn C57BL/6 mice grew into neurospheres. The immunostaining results showed that these neurospheres were double-positive for Sox-2 and Nestin (Fig. 1 D). Cultured in differentiation medium for 7 days, the monolayer NSCs differentiated into astrocytes (GFAP^+^/DAPI), neurons (NF-M^+^/DAPI) and oligodendrocytes (NG2^+^/DAPI) (Fig. 1 E), indicating the multipotent differentiation of the cultured NSCs.

To achieve AD cell model, the cultured NSCs were transfected with GFP and APP-GFP lentiviral vectors, respectively. As shown in Fig. 1 F, green fluorescence was observed both in GFP-NSCs and APP-NSCs under a fluorescence microscope. The RT-PCR and Western blot results indicated that both mRNA and protein of APP and Aβ_42_ were over-expressed in APP-NSCs compared with that in GFP-NSCs (Fig. 1 G-I). These results indicated that an AD cell model (APP-NSCs) overexpressing Aβ_42_ proteins was established successfully.

### The effects of the three active ingredients on cell viability and damages of APP-NSCs

**Fig. 2:**
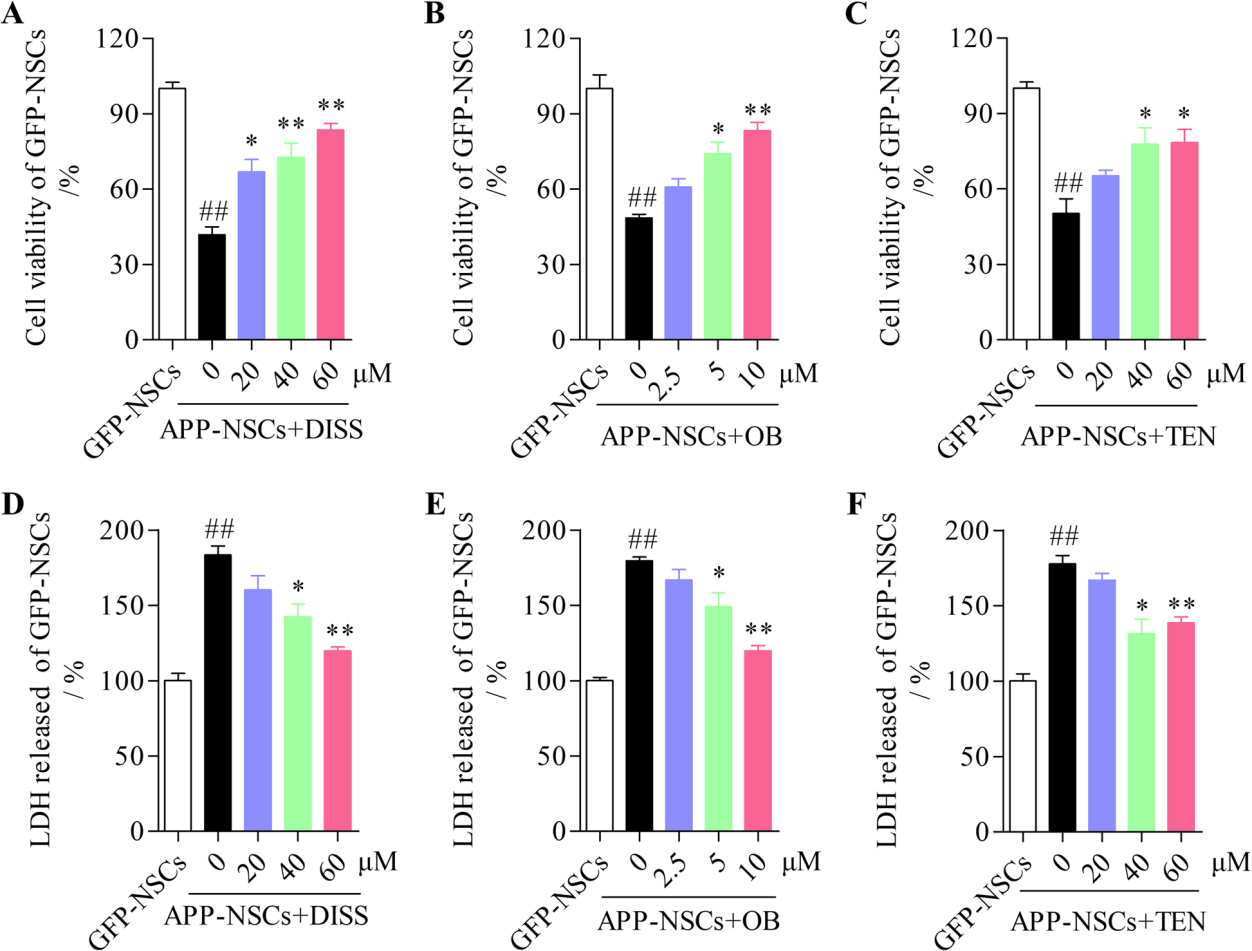
The three active ingredients protected APP-NSC from death and damage. The monolayer cells were cultured in proliferation medium for 24 h, exposed to DISS (20; 40 and 60 μM), OB (2.5; 5 and 10 μM) and TEN (20; 40 and 60 μM), respectively for another 24 h. Then the MTT and LDH assays were carried out. (A-C) The viability of APP-NSCs administrated with DISS (A), OB (B) or TEN (C) were measured by the MTT assay. (D-F) The LDH leakage of APP-NSCs exposed to DISS (D), OB (E) or TEN (F) were detected by the LDH kit. ##*P* < 0.01 vs. GFP-NSCs group; *P < 0.05; **P < 0.01 vs. APP-NSCs group. Data are represented as mean ± sem. This experiment was performed three times. Data were analyzed by one-way ANOVA

The MTT results showed that the cell viability in APP-NSCs was remarkably lower than that in GFP-NSCs (Fig. 2 A-C, ##*P* < 0.01 vs. GFP-NSCs). After exposure to different compounds, the cell viability was does-dependently increased (Fig. 2 A-C, **P < 0.01 in 40 and 60 μM of DISS; **P < 0.01 in 10 μM of OB; *P < 0.05 in 60 μM of TEN). Similarly, the three active compounds could all recused the excessive leakage of LDH in APP-NSCs (Fig. 2 D-F, **P < 0.01 in 60 μM of DISS; **P < 0.01 in 10 μM of OB; **P < 0.01 in 60 μM of TEN). These findings indicated that the three active compounds could protect APP-NSCs from death and damages. The optimal concentrations including 60 μM of DISS, 10 μM of OB and 60 μM of TEN were used in the following experiments in vitro.

### The effects of the three active ingredients on proliferation of APP-NSCs

**Fig. 3:**
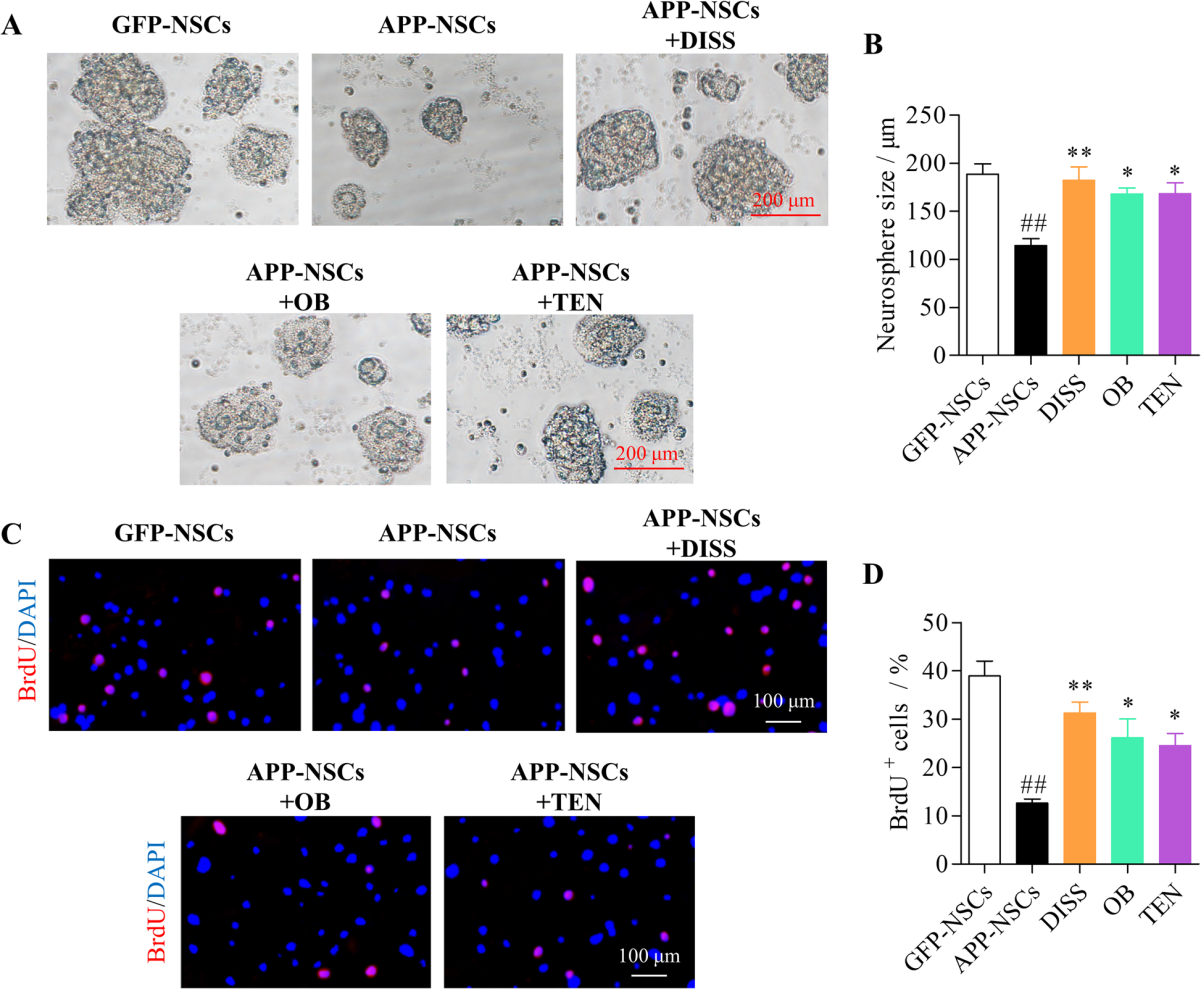
The effects of the three ingredients on the proliferation of APP-NSCs. APP-NSCs were cultured in proliferation medium, and exposed to DISS (60 μM), OB (10 μM), and TEN (60 μM) for 7 days, respectively. The neurospheres images were captured and the diameters of neurospheres were measured using by Image J software. (A) The representative micrographs of neurospheres in different group. Scale bar = 200 μm; (B) Quantitative analysis of diameters of neurospheres in different group; (C) The representative micrographs of BrdU^+^ cells; scale bar = 100 μm. (D) Quantitative analysis of BrdU^+^ cells. ##*P* < 0.01 vs. GFP-NSCs group; *P < 0.05; **P < 0.01 vs. APP-NSCs group. Data are represented as mean ± sem. This experiment was performed three times. Data were analyzed by one-way ANOVA

As shown in Fig. 3, the size of neurospheres in APP-NSCs group were remarkably decreased compared with that in GFP-NSCs group (Fig. 3 A-B, *##P* < 0.01 vs. GFP-NSCs group). Whereas, APP-NSCs treated with 60 μM of DISS, 10 μM of OB or 60 μM of TEN displayed enlarged diameters compared with that in APP-NSCs group (Fig. 3 A-B, DISS: **P < 0.01; OB: *P < 0.05; TEN: *P < 0.05 vs. APP-NSCs). Similarly, there were less BrdU-positive cells in APP-NSCs group (Fig. 3 C-D, *##P* < 0.01 vs. GFP-NSCs group), but more BrdU^+^ cells were observed in the three active ingredients treatment groups (Fig. 3 C-D, DISS: **P < 0.01; OB: *P < 0.05; TEN: *P < 0.05 vs. APP-NSCs). These results demonstrated that DISS, OB and TEN could partially rescued proliferation deficits of APP-NSCs. DISS possessed the optimal effect among the three compounds of PT.

### The effects of the three active ingredients on migration of APP-NSCs

**Fig. 4:**
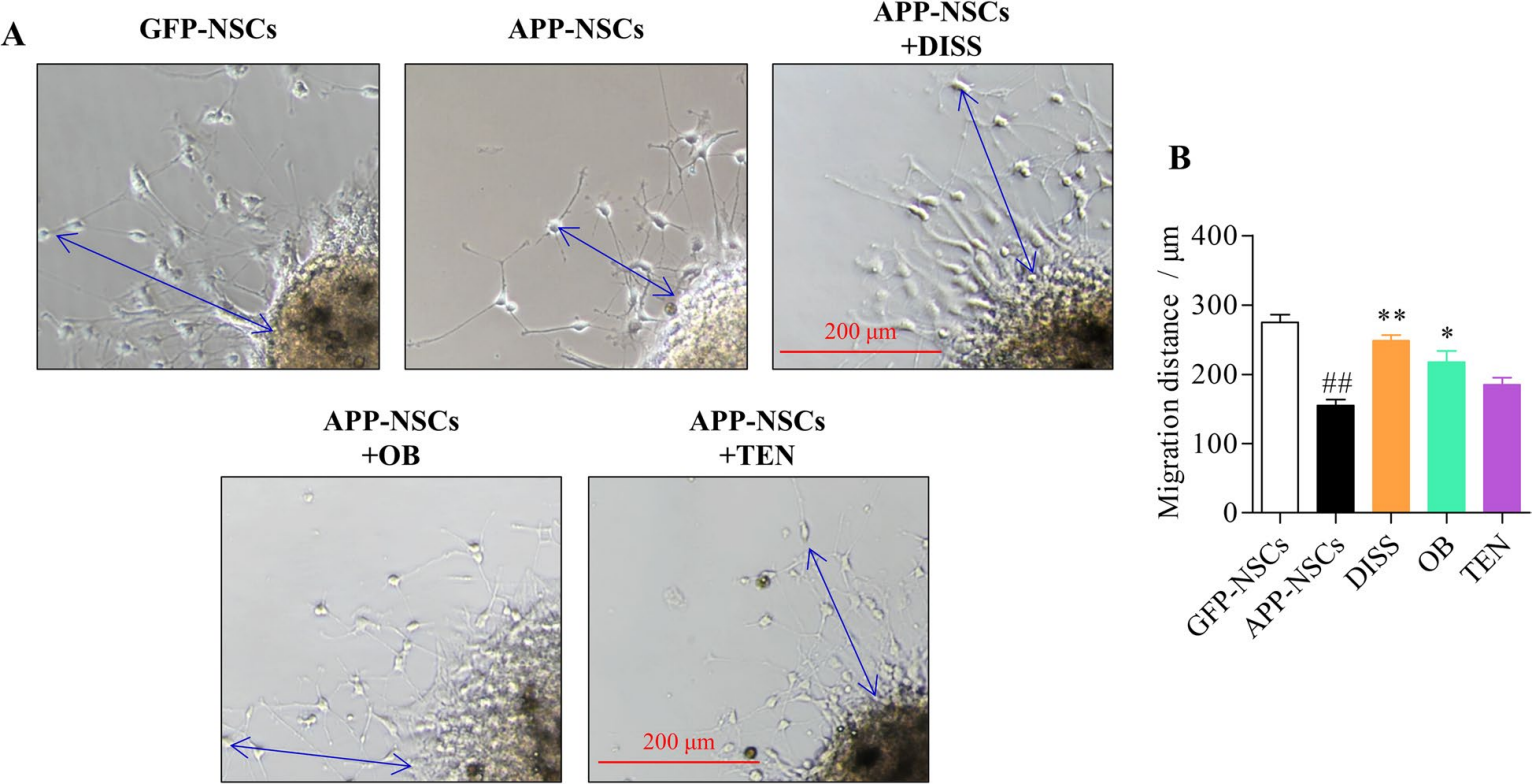
The effects of the three ingredients on the migration of APP-NSCs. APP-NSCs were cultured in differentiation medium, and exposed to DISS (60 μM), OB (10 μM), and TEN (60 μM) for 7 days, respectively. The neurospheres images were captured and the migration distance of neural cells from the edge of the neurospheres were measured using by Image J software. (A) The representative micrographs of neurospheres in different group. Scale bar = 200 μm; (B) Quantitative analysis of distance of neural cells from the neurosphere. ##*P* < 0.01 vs. GFP-NSCs group; *P < 0.05; **P < 0.01 vs. APP-NSCs group. Data are represented as mean ± sem. This experiment was performed three times. Data were analyzed by one-way ANOVA

NSCs about 200 μm in diameter were exposed to 60 μM of DISS, 10 μM of OB and 60 μM of TEN for 3 days, respectively, followed by migration assays. The neural cells were observed to migrate radially outward from the emerge of the neurospheres (Fig. 4 A). The migration distances of APP-NSCs were strikingly shorter as compared with that in GFP-NSCs (Fig. 4 A-B, ##*P* < 0.01). DISS (60 μM) and OB (10 μM) treatment prominently extended the migration distances (Fig. 4 A-B, **P < 0.01 in DISS; *P < 0.01 in OB vs. APP-NSCs group). However, TEN could not increase the migration distances of APP-NSCs (*P > 0.05 vs. APP-NSCs group). These results indicated that DISS (60 μM) or OB (10 μM) treatments partially recovered migration of APP-NSCs.

### The effects of the three active ingredients on differentiation of APP-NSCs

**Fig. 5:**
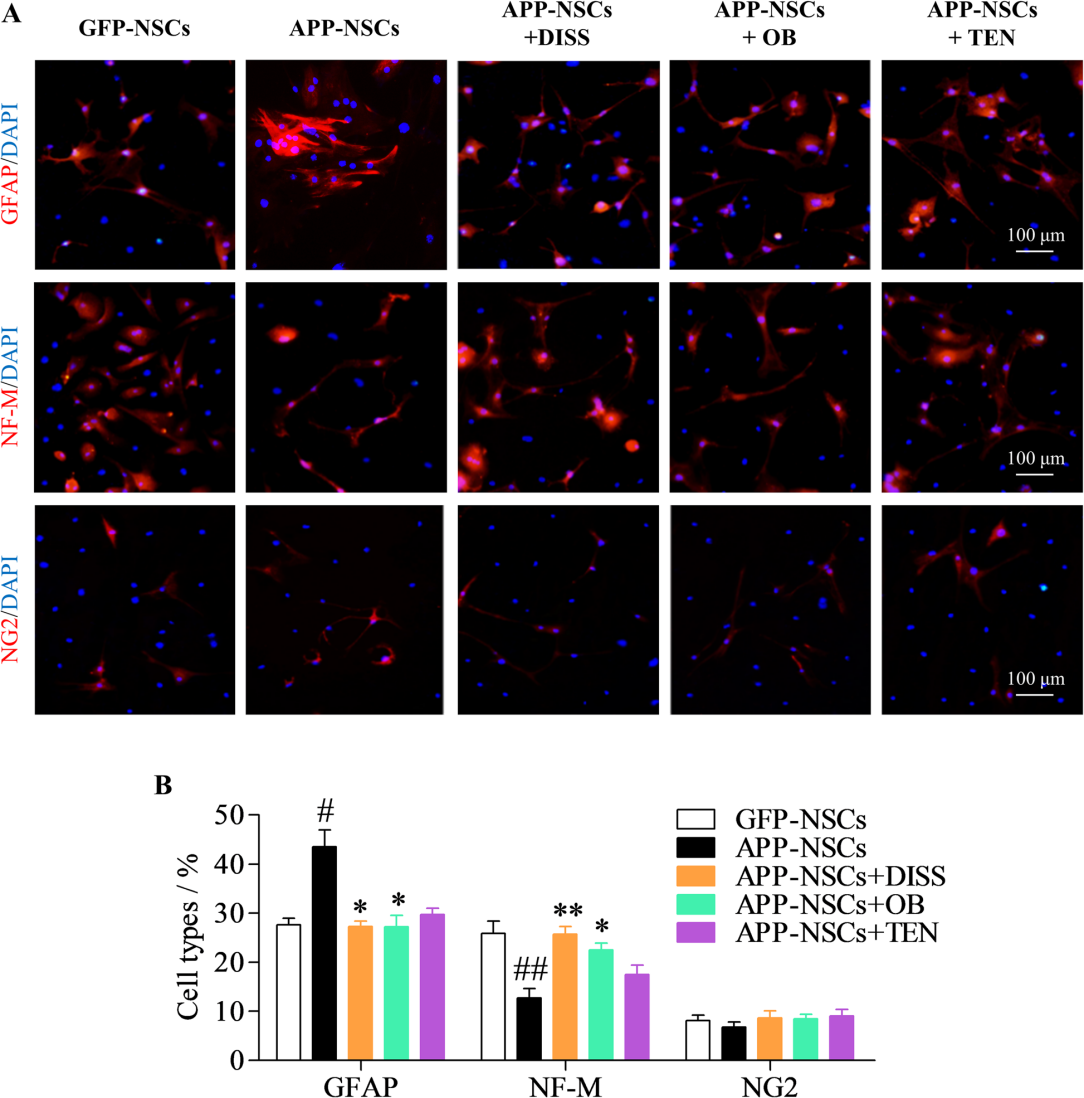
The effects of the three active ingredients on the differentiation of APP-NSCs. APP-NSCs were cultured in differentiation medium, exposed to DISS (60 μM); OB (10 μM), and TEN (60 μM) for 7 days, then the cell types were detected using by IF analysis against GFAP, NF-M and NG2 antibody, respectively. (A) The representative micrographs of APP-NSCs differentiated into astrocytes (GFAP^+^); neuronal cells (NF-M^+^) and oligodendrocytes (NG2^+^). Scale bar = 100 μm; (B) The quantitative analysis of astrocytes, neurons, and oligodendrocytes as in A. #*P* < 0.05, ##*P* < 0.01 vs. GFP-NSCs group; *P < 0.05; **P < 0.01 vs. APP-NSCs group. Data are represented as mean ± sem. This experiment was performed three times. Data were analyzed by one-way ANOVA

As shown in Fig. 5 A-B, the percentage of astrocytes (GFAP^+^/DAPI) in APP-NSCs group increased (Fig. 5 A-B, #*P* < 0.05 vs. GFP-NSCs), the proportion of neurons (NF-M^+^/DAPI) decreased (Fig. 5 A-B, ##*P* < 0.01 vs. GFP-NSCs), and the oligodendrocyte (NG2^+^/DAPI) percentage was not markedly altered (Fig. 5 A-B, *P* > 0.05 vs. GFP-NSCs), indicating differentiation defects of APP-NSCs. The astrocyte percentage in DISS (60 μM), OB (10 μM) and TEN (60 μM) treatment groups were significantly decreased (Fig. 5 A-B, **P < 0.01 in DISS; *P < 0.05 in OB; *P < 0.05 in TEN vs. APP-NSCs), and the neuron percentage in DISS (60 μM) and OB (10 μM) treatment groups were notably increased (Fig. 5 A-B, **P < 0.01 in DISS; *P < 0.05 in OB vs. APP-NSCs). However, TEN (60 μM) has no significant effects on the differentiation of APP-NSCs (Fig. 5 A-B, *P* > 0.05 vs. APP-NSCs). These results demonstrated that DISS (60 μM) and OB (10 μM) treatment could effectively rescue the differentiation defects of APP-NSCs. Moreover, DISS exerted optimal effect to promoting neuronal differentiation of APP-NSCs, thus DISS was used in the following in vivo experiments.

### DISS rescued cognitive deficits in adult APP/PS1 mice

**Fig. 6:**
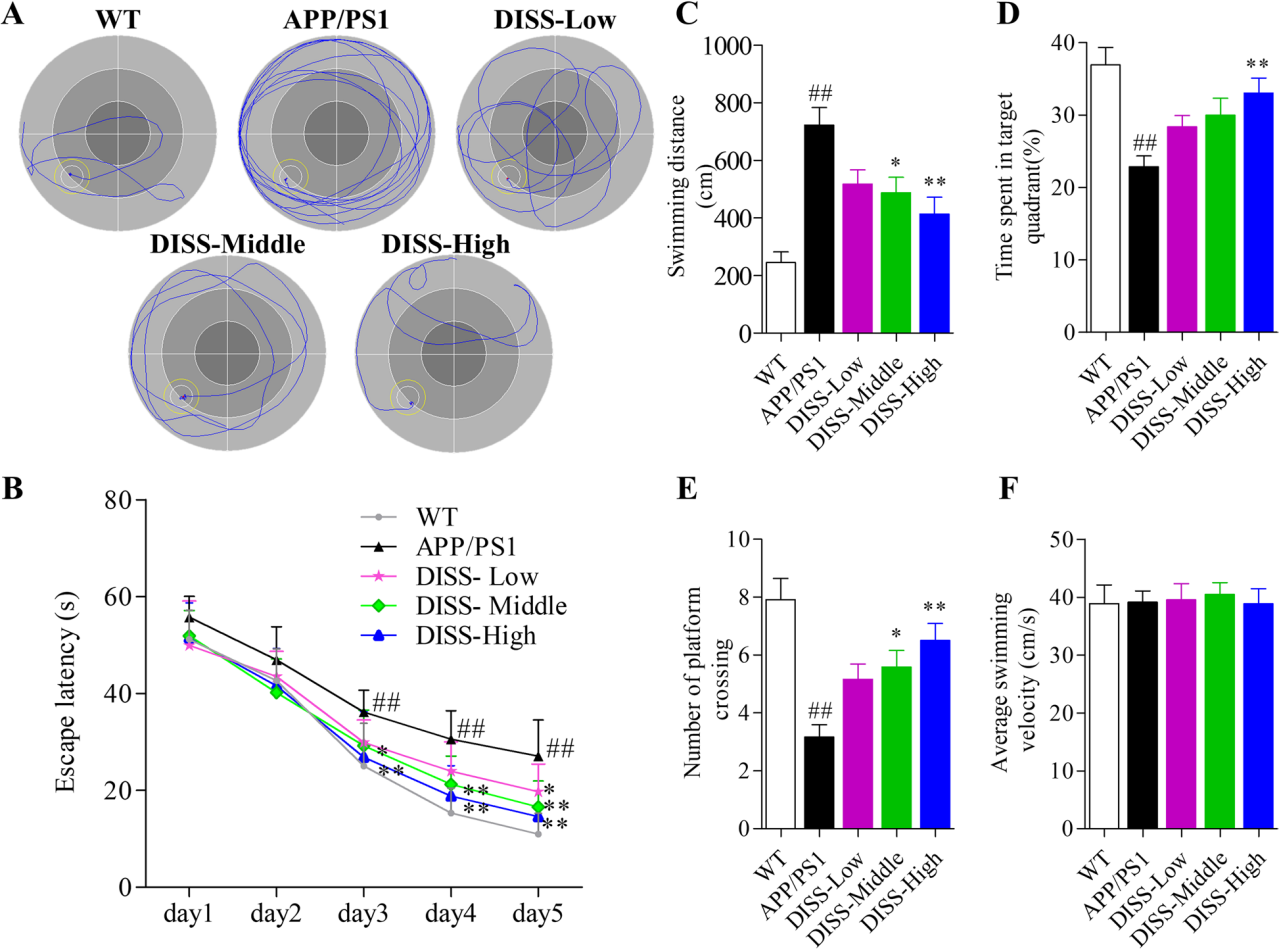
DISS rescued cognitive deficits in APP/PS1 mice. After 4-weeks treatment, MWM test was carried out to examine the cognitive function of mice in each group. (A) The representative swimming paths of each group in the training trails at day 5; (B) The escape latency in the training trials; (C) The swimming distances before crossing the platform; (D) The time spent in the target quadrant; (E) The times of platform crossing; (F) The swimming velocity in the probe trails. #*P* < 0.05; ##*P* < 0.01 vs. WT group; *P < 0.05; **P < 0.01 vs. APP/PS1 group. Data are represented as mean ± sem. *n* = 10 mice in each group. The escape latencies in different groups were analyzed by repeated-measures two-way ANOVA, and the other data were analyzed by one-way ANOVA

MWM test was carried out to assess the learning and memory ability of mice in different groups. The representative swimming paths in the training trials at day 5 were as shown in Fig. 6 A. Mice in APP/PS1 group exhibited no preference towards the target quadrant with longer escape latency (Fig. 6 B, ##*P* < 0.01 vs. WT group from day 3 to day 5), longer swimming distances (Fig. 6 C, ##*P* < 0.01 vs. WT group), shorter time spent in the target quadrant (Fig. 6 D, ##*P* < 0.01 vs. WT group), and fewer platform crossing numbers (Fig. 6 E, ##*P* < 0.01 vs. WT group) compared with WT group, indicating severe cognitive damages in APP/PS1 mice. Fortunately, DISS treatment dose-dependently ameliorated the cognitive deficits of APP/PS1 mice (Fig. 6 A-E, *P < 0.05, **P < 0.01 vs. APP/PS1 group). There was no remarkable difference among the swimming velocity in each group (Fig. 6 F), which indicated that DISS had no significant effects on motor ability of mice.

### DISS ameliorated pathological deficits of hippocampal neurons in adult APP/PS1 mice

**Fig. 7:**
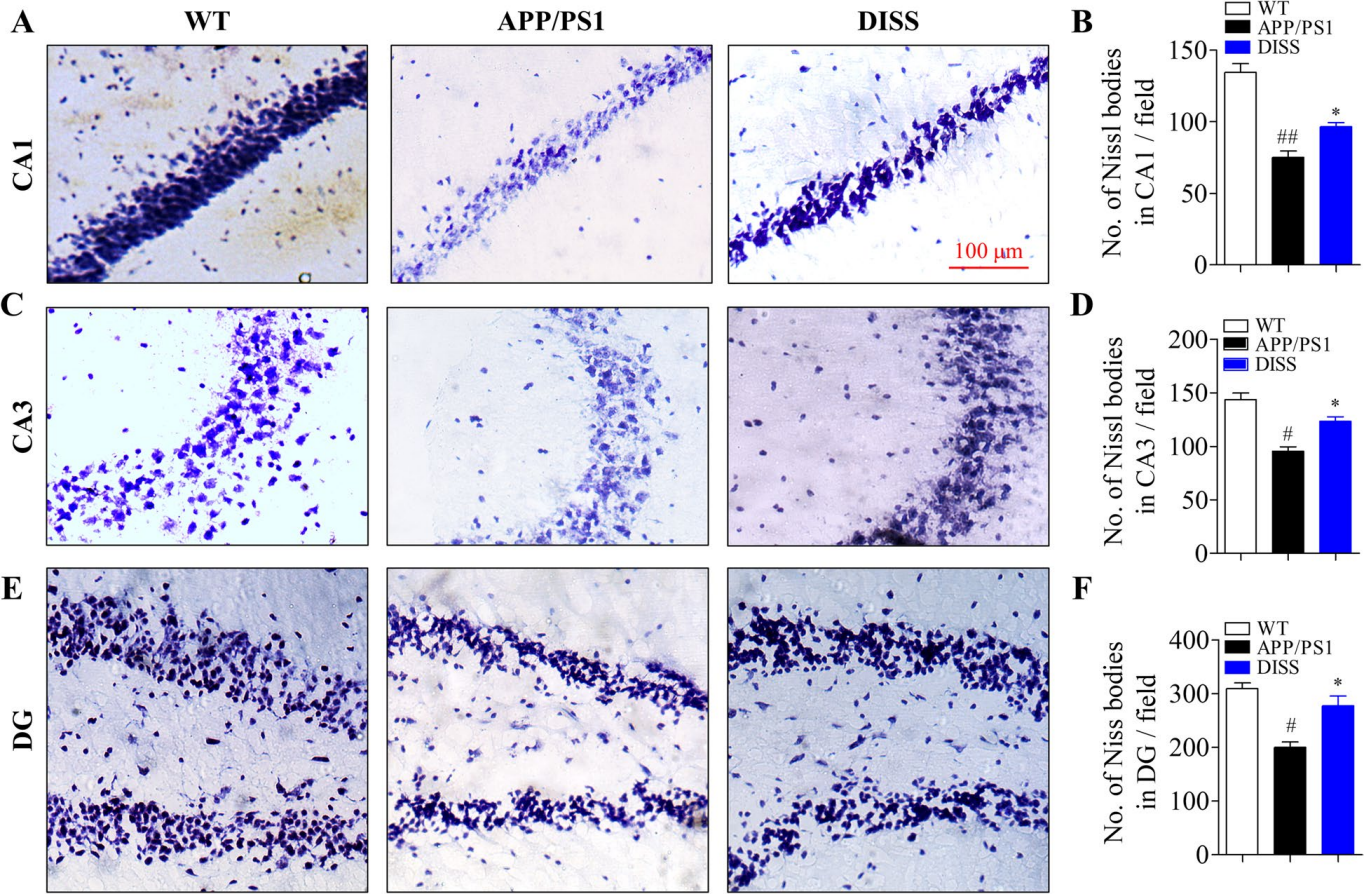
DISS rescued hippocampal neurons damages in APP/PS1 mice. Nissl staining analysis was carried out to detect the damages of hippocampal neurons. (A, C and E) Representative images of Nissl staining of the hippocampal CA1 (A), CA3 (C) and DG (E) regions, respectively. Scale bar = 100 μm. (B, D and F) Quantitative analysis of Nissl bodies in the hippocampal CA1 (B), CA3 (D) and DG (F) regions, respectively. #*P* < 0.05; ##*P* < 0.01 vs. WT group; *P < 0.05 vs. APP/PS1 group. Data are represented as mean ± sem. *n* = 6 mice in each group; six slices per animal analyzed by one-way ANOVA

Nissl body, a representative structure of neurons, consists of a host of rough endoplasmic reticulum and free ribosomes, and plays a vital role in protein synthesis. Thus, it can be served as an indicator reflecting the size, number, morphology, location and survival of neurons [39, 40]. A large number of Nissl-positive cells can be observed in the hippocampal CA1, CA3 and DG areas in TW group (Fig. 7 A-F). However, the number of Nissl bodies were severely decreased in the hippocampal CA1, CA3 and DG areas in the brain of APP/PS1 mice (Fig. 7 A-F, CA1: ##*P* < 0.01; CA3: #*P* < 0.05; DG: #*P* < 0.05 vs. WT group). Whereas, administration with DISS (20 mg/kg) strikingly elevated the number of Nissl bodies compared to APP/PS1 group (Fig. 7 A-F, *P < 0.05 vs. APP/PS1 group). These results demonstrated that DISS effectively reduced the pathological damages of hippocampal neurons in APP/PS1 mice.

### DISS enhanced hippocampal NSCs proliferation in adult APP/PS1 mice

**Fig. 8:**
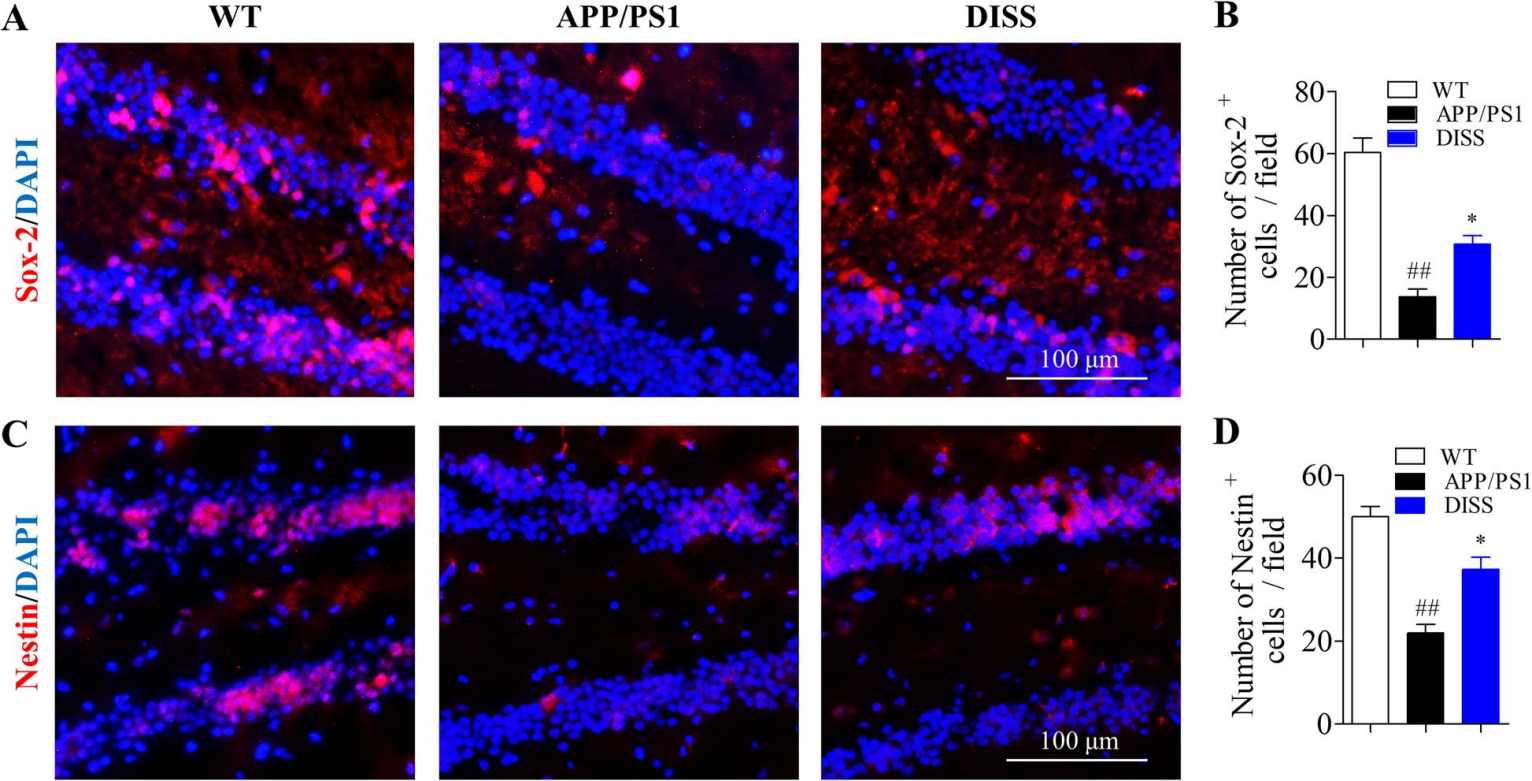
DISS promoted the proliferation of hippocampal NSCs in APP/PS1 mice. IF analysis against Sox-2 and Nestin was carried out to detect the NSCs in the hippocampus of APP/PS1 mice. (A) Representative immunostaining images of Sox-2^+^ cells in the hippocampal DG areas. (B) Quantitative analysis of Sox-2^+^ cells. (C) Representative immunostaining images of Nestin^+^ cells in the hippocampal DG areas. (D) Quantitative analysis of Nestin^+^ cells. ##*P* < 0.01 vs. WT group; *P < 0.05 vs. APP/PS1 group. Scale bar = 100 μm. Data are represented as mean ± sem. n = 6 mice in each group; six slices per animal analyzed by one-way ANOVA

IF staining assays against Sox-2 and Nestin were carried out to detect the NSCs in the hippocampus. The immunostaining results showed that NSCs labeled by Sox-2 in the DG areas in APP/PS1 group were obviously lessened compared to WT group (Fig. 8 A-B, ##*P* < 0.01 vs. WT group). However, DISS (20 mg/kg) treatment for 4 weeks considerably augmented the number of Sox-2-positive cells (Fig. 8 A-B, *P < 0.01 vs. APP/PS1 group). Similarly, fewer Nestin-positive cells were observed in hippocampal DG regions in APP/PS1 group (Fig. 8 C-D, ##*P* < 0.01 vs. WT group), but more Nestin-positive cells were detected in the DISS treatment group (Fig. 8 C-D, *P < 0.05 vs. APP/PS1 group). These results demonstrated that DISS could promote the NSCs proliferation in the hippocampal DG regions in APP/PS1 mice.

### DISS increased the number of neurons in adult APP/PS1 mice

**Fig. 9:**
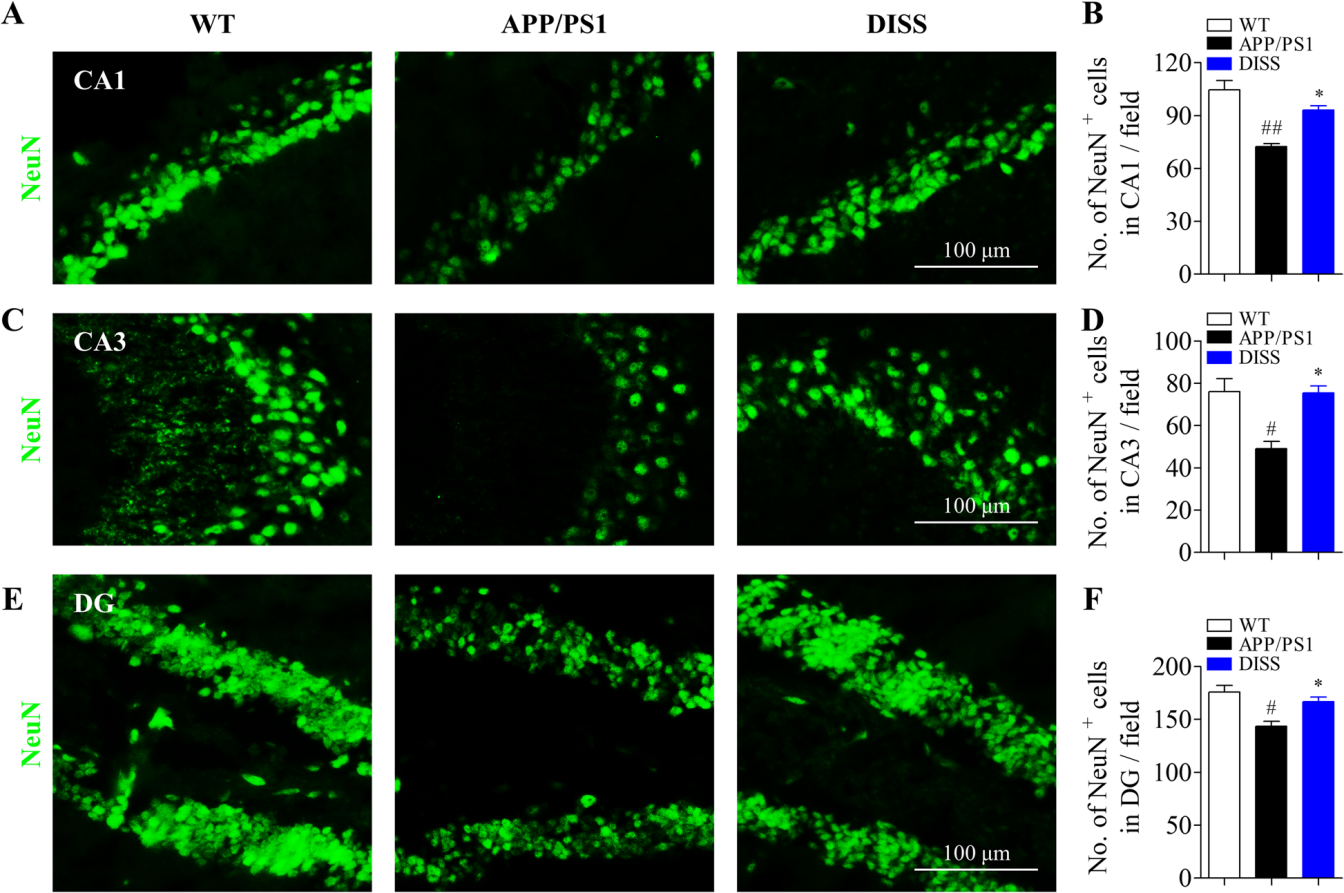
DISS promoted the neuronal differentiation of hippocampal NSCs in APP/PS1 mice. IF analysis against NeuN was carried out to detect the mature NSCs in the hippocampus of APP/PS1 mice. (A, C and E) Representative immunostaining images of NeuN^+^ cells in the hippocampal CA1 (A), CA3 (C) and DG (E) regions, respectively. Scale bar = 100 μm. (B, D and F). Quantitative analysis of NeuN^+^ cells in the hippocampal CA1 (B), CA3 (D) and DG (F) regions, respectively. #*P* < 0.05; ##*P* < 0.01 vs. WT group; *P < 0.05 vs. APP/PS1 group. Data are represented as mean ± sem. n = 6 mice in each group; 6 slices per animal were analyzed by one-way ANOVA

Mature neurons were detected using by IF staining against NeuN. The results showed that NeuN-positive cells in the hippocampal CA1, CA3 and DG regions were strikingly reduced in APP/PS1 group (Fig. 9 A-F, CA1: ##*P* < 0.01; CA3: #*P* < 0.05; DG: #*P* < 0.05 vs. WT group), indicating neurogenesis deficits in APP/PS1 mice. Fortunately, DISS (20 mg/kg) treatment for 4 weeks effectively augmented the number of mature neurons (NeuN^+^/DAPI) (Fig. 9 A-F, *P < 0.05 vs. APP/PS1 group), indicating a positive stimulation of DISS on hippocampal neurogenesis in APP/PS1 mice.

## Discussion

In adult brain, hippocampal neurogenesis takes place throughout the whole life and plays critical roles in sustaining and restoring the cognitive function [10, 41]. However, compromised neurogenesis was observed in both AD patients and AD animals. Emerging evidence demonstrates that hippocampal neurogenesis is impaired prior to the onset of AD pathology, and contributes to the initiation and development of neuropathology in AD [42–45]. In the present study, we evaluated the effects of three active chemical constituents (DISS, OB and TEN) of PT on the survival, proliferation, migration and neuronal differentiation of APP-NSCs in vitro, and further identified the potential active chemical of PT to promote neurogenesis in vivo. We demonstrated that DISS cloud stimulate the proliferation, migration and neuronal differentiation of APP-NSCs, and rejuvenate hippocampal neurogenesis to promote the cognitive function in adult APP/PS1 mice.

The amyloid hypothesis posits that the excessive accumulation of Aβ leads the progressive dysfunction and degeneration of neurons in AD [32, 38, 46, 47]. Furthermore, it has been revealed that Aβ_42_ oligomers compromise the mtDNA repair, shift NSCs differentiation towards an astrocytic lineage [48], as well as impair the migratory capacity of NSCs [49]. Considering the vital role of Aβ in the progress of AD, we established an AD cell model using by lentiviral transfection of mutant APP gene into NSCs named as APP-NSCs. Consistent with our previous studies [29, 30], the APP-NSCs continuously express APP mutant gene disturbing the APP metabolism such that lead to high levels of Aβ in the cells (Fig. 1 D-E), which ultimately impaired the cell viability, proliferation, and differentiation of APP-NSCs.

The DISS, OB and TEN are the main constituents of PT, which is a famous traditional Chinese medicine with multiple neuroprotective effects on central nervous system diseases. DISS has been revealed to improve the cell viability and protect SH-SY5Y cells from apoptosis induced by glutamate and H_2_O_2_ [24, 25], as well as ameliorate hippocampal plasticity in rats suffering from chronic mild stress [50]. OB has been reported to mitigate the cognitive impairments in APP/PS1 mice [22], aging rats induced by D-galactose [23], as well as LPS-injured rats [51]. The suppressing effects of OB on neuroinflammation, apoptosis, oxidative stress and Aβ pathology may relate to NF-κB/p65, Nrf-2/HO-1, Bcl-2/Bax/caspase-9 signaling pathways [22, 23, 51]. TEN is a metabolite of OB [26], and oral administration of TEN markedly increased the learning and memory ability in AD animals by anti-apoptosis [27], anti-neuroinflammation [28] and reduction of Aβ secretion [16, 52–54]. The activities of anti-apoptosis, anti-neuroinflammation, anti-oxidative stress, anti-Aβ pathology are beneficial for hippocampal neurogenesis, however, there is little literature about whether these compounds could rescue the damages of hippocampal neurogenesis in APP/PS 1 mice.

In present study, we demonstrated that DISS, OB and TEN could all enhance the cell viability (Fig. 2), rescue the dysfunction of proliferation (Fig. 3) of APP-NSCs. Interestingly, only DISS and OB were observed to facilitate the migration (Fig. 4) and neuronal differentiation of APP-NSCs (Fig. 5). Hippocampal neurogenesis is a multistep process involving NSC proliferation, migration, neuronal differentiation, neuronal mature and integration into the preexisting neural circuits [8, 55, 56]. The proliferation, migration and neuronal differentiation capable of NSCs in vitro can reflect the neurogenesis ability in vivo, and compounds which can positively modulate these processes in vitro likely to strength the neurogenesis in vivo [14, 32]. Among the three compounds investigated in present study, DISS displayed the optimal potence to enhance the neurogenesis in vitro, thus, we chose DISS to investigate whether it could stimulate hippocampal neurogenesis in the brain of adult APP/PS1 mice.

The APP/PS1 mouse is a common rodent model to study the neuropathology and therapeutic strategies of AD. The APP/PS1 mouse expresses both human APPswe and PS1∆E9 mutated genes, which alter APP metabolism and result in overproduction of Aβ with age, and Aβ plaques can be observed at about 9 months of age [57–59]. Our findings indicated that gavage administration of DISS (20 mg/kg) for 4 weeks strikingly mitigated the learning and memory dysfunctions of APP/PS1 mice (Fig. 6), increased the amount of Nissl bodies (Fig. 7). In addition, DISS (20 mg/kg) treatment for 4 weeks remarkably fostered hippocampal NSCs proliferation and neuronal differentiation presented by increased number of Sox-2^+^, Nestin^+^ and NeuN^+^ cells in the hippocampus (Fig. 8 and Fig. 9). Sox-2 and Nestin are the biomarkers of NSCs, and NeuN is the biomarker of mature neurons. These data indicated that DISS could promote hippocampal neurogenesis in APP/PS1 mice. It has been revealed that DISS can increase the expression of BDNF and promoted phosphorylation of CREB via the CaMKII and ERK1/2 pathway [25], which beneficial for hippocampal neurogenesis [60, 61].

In summary, our findings indicated that DISS is the potential active ingredient of PT to promote neurogenesis, and DISS has the property to rejuvenate hippocampal neurogenesis to mitigate the learning and memory damages in adult APP/PS1 mice. However, the molecular mechanisms underlying the neurogenesis medicated by DISS in AD pathology still need to be clarified in the future.

## Conclusions

Our findings demonstrated DISS is the constituent of PT that triggers the most potent increase of hippocampal neurogenesis in APP/PS1 transgenic mice.

## Supplementary Information

**Additional file 1.**

## Data Availability

The datasets used and/or analyzed during the current study available from the corresponding author upon reasonable request.

## Abbreviations

AD: Alzheimer’s disease
DISS: 3;6′-Disinapoyl sucrose
OB: Onjisaponin B
TEN: Tenuifolin
PT: *Polygala tenuifolia*
NSCs: Neural stem cells
APP-NSCs: NSCs transfected with amyloid precursor protein (APP) gene
MTT: 3-(4,5- Dimethylthiazol-2-yl)-2,5-diphenyltetrazolium bromide
LDH: Lactate dehydrogenase
IF: Immunofluorescence
BrdU: 5-Bromo-2′-deoxyuridine
MWM: Morris water maze
NFTs: Neurofibrillary tangles
SPs: Senile plaques
Aβ: Amyloid beta
FBS: Fetal bovine serum
EGF: Epidermal growth factor
bFGF: Basic fibroblast growth factor
GFAP: Glial fibrillary acidic protein
NF-M: Intermediate neurofilament
Sox-2: Sex-determining region Y-box 2
NG-2: Neural/glial antigen-2
NeuN: Neuron-specific nuclear protein
WT: Wild-type
NEAA: Non-essential amino acid
GFP: Green fluorescent protein
PFA: Paraformaldehyde
