# Supplementary figures and images for: Active constituent of Polygala tenuifolia attenuates cognitive deficits by rescuing hippocampal neurogenesis in APP/PS1 transgenic mice
Source: BMC Complement Med Ther. 2021 Oct 25;21:267. doi: 10.1186/s12906-021-03437-5 (PMC8543956; doi:10.1186/s12906-021-03437-5)

Western blot figures


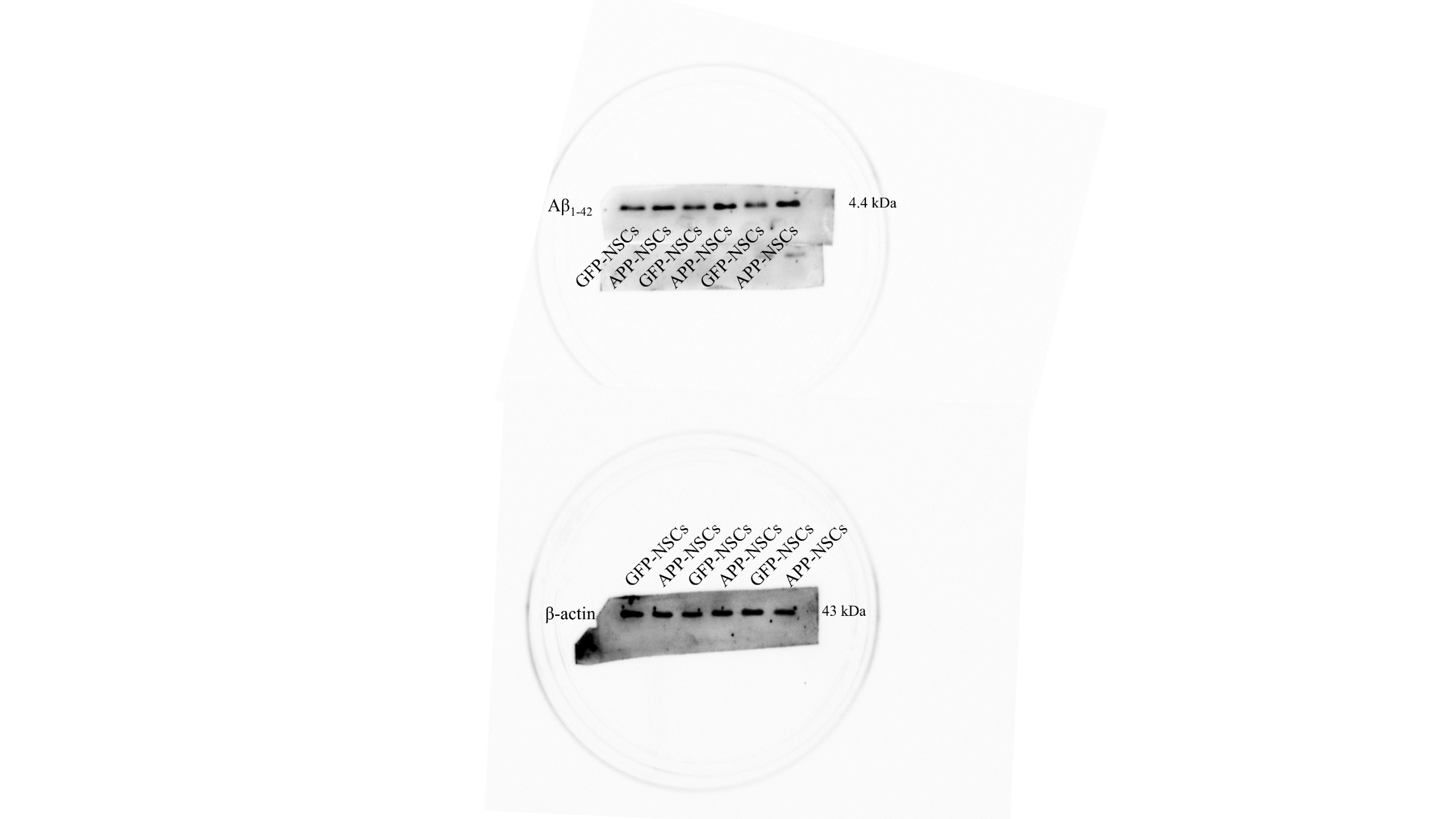


RT-PCR figures


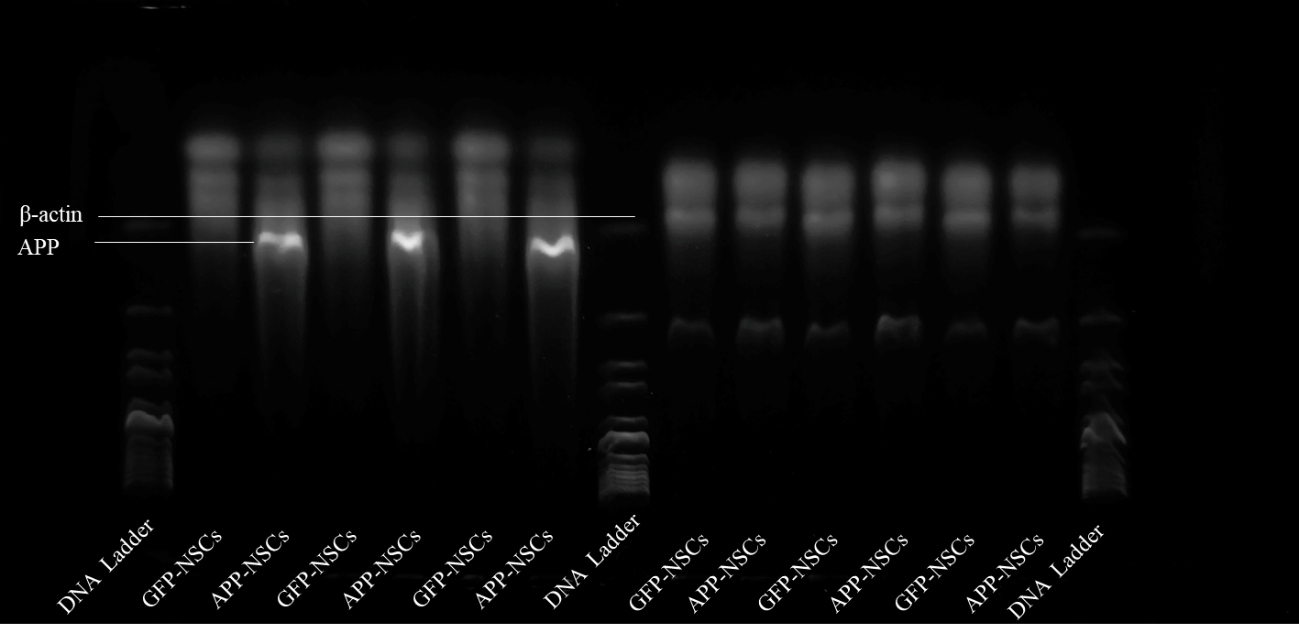

Supplement: Supplementary file 1 — Additional file 1. [file 12906_2021_3437_MOESM1_ESM.docx]
